# The Effects of Resveratrol Treatment on Bcl-2 and Bax Gene Expression in Endometriotic Compared with Non-Endometriotic Stromal Cells

**DOI:** 10.18502/ijph.v49i8.3900

**Published:** 2020-08

**Authors:** Roya KOLAHDOUZ-MOHAMMADI, Ali-Akbar DELBANDI, Sepideh KHODAVERDI, Soheila AREFI, Tahereh ARABLOU, Farzad SHIDFAR

**Affiliations:** 1. Department of Nutrition, School of Public Health, Iran University of Medical Sciences, Tehran, Iran; 2. Department of Immunology, School of Medicine, Iran University of Medical Sciences, Tehran, Iran; 3. Immunology Research Center, Institute of Immunology and Infectious Diseases, Iran University of Medical Sciences, Tehran, Iran; 4. Endometriosis Research Center, Iran University of Medical Sciences, Tehran, Iran; 5. Bahman Hospital Infertility Center, Tehran, Iran; 6. Genetics and In Vitro Assisted Reproductive (GIVAR) Center, Erfan Hospital, Tehran, Iran

**Keywords:** Endometriosis, Resveratrol, Apoptosis, Gene expression, Antioxidant

## Abstract

**Background:**

We aimed to examine resveratrol effects on gene expression of Bcl-2, Bax and Bcl-2/Bax ratio in endometrial stromal cells derived from women with and without endometriosis.

**Methods:**

Endometrial tissues were obtained from 40 endometriotic patients and 15 non-endometriotic controls undergoing laparoscopic surgery or hysterectomy in the gynecology ward of Rassoul Akram Hospital, Tehran, Iran from 2015 to 2017. After the enzymatic digestion, eutopic (EuESCs) and ectopic (EESCs) endometrial stromal cells from patients with endometriosis as well as endometrial stromal cells from non-endometriotic controls (CESCs) were treated with or without resveratrol (100 μM) and the levels of Bcl-2, Bax and Bcl-2/Bax gene expression ratio in the cells from all origins were examined at 6, 24 and 48 h post-treatment by real-time PCR.

**Results:**

Resveratrol treatment increased Bcl-2 expression in CESCs at 24 and 48 h and in EuESCs at 48 h (*P*<0.05), but had no significant effects on the expression of this gene in EESCs. On the other hand, resveratrol treatment increased Bax expression in EuESCs at 6 h and decreased its expression in EESCs at 48 h (*P*<0.05). Regarding the Bcl-2/Bax gene expression ratio, resveratrol treatment increased Bcl-2/Bax gene expression ratio in CESCs and EuESCs at 48 h (*P*<0.01). However, this treatment had no significant differential effect on Bcl-2 and Bcl-2/Bax gene expression ratio between CESCs and EuESCs at 48 h.

**Conclusion:**

Resveratrol treatment significantly increased Bcl-2/Bax gene expression ratio in EuESCs and CESCs but had no significant effect in EESCs.

## Introduction

Endometriosis, defined as the presence of viable endometrial-like tissues, including glands and stroma outside the uterine cavity, is associated with pelvic pain, dysmenorrhea and infertility (1). Endometriosis is histologically categorized into three distinct forms: ovarian endometriosis (endometrioma), peritoneal endometriosis and deeply infiltrating endometriosis (DIP) (2). Regardless of being a common and serious health problem in women, the exact etiology of endometriosis remains unclear. Based on published studies, retrograde menstruation (Sampson theory) of endometrial cells with subsequent implantation and growth of viable endometrium within the peritoneal cavity have an important role in triggering and developing the disease (1). Ample evidence indicates that the endometrial cells from women with and without endometriosis have different capacities to invade apoptosis (3), proliferate, implant and survive in the ectopic locations (4).

Apoptosis in the eutopic endometrium of endometriotic patients was lower compared to non-endometriotic patients. Moreover, ectopic endometrium of endometriotic patients showed less apoptosis compared with their eutopic counterparts (3, 5). Besides, early studies provided conflicting data concerning the difference in anti-apoptotic B-cell lymphoma/leukemia-2 (Bcl-2) and pro-apoptotic Bax gene expression between normal endometrial tissue and eutopic and/or ectopic endometrial tissue in endometriotic women (6). Therefore, manipulation of programmed cell death processes by enhancing endogenous or exogenous apoptotic stimulants in the lesions could be used to treat endometriosis.

In recent years, naturally occurring phytochemicals such as resveratrol have gained considerable attention because of their health-promoting properties (7). Resveratrol (trans-3,4′,5-trihydroxystilbene, C14H12O3) is a phytoalexin synthesized by plants in response to fungal infection or exposure to ultraviolet light and is present in dietary products such as grapes and red wine (8). In the last few decades, resveratrol and other stilbenes have gained considerable attention, particularly in invitro and preclinical studies due to their antioxidant, anti-inflammatory, anti-neoplastic and other health-promoting effects (9).

Therapeutic effects of resveratrol on endometriosis was first described by Bruner-Tran et al in 2011 (10). From that moment, the interest in this natural compound has grown extremely and many researchers tried to find protective mechanisms of resveratrol on endometriosis. Resveratrol controls the development of endometriosis by suppression of proliferation in endometriotic lesions (10, 11), induction of apoptosis (10, 11), reduction of inflammation (12, 13), angiogenesis (12, 14, 15) and oxidative stress (16). With regard to apoptosis, previous studies examined the effects of resveratrol on experimental endometriotic mice models gained conflicting results (10, 11, 17). However, the effect of resveratrol treatment on apoptosis in endometriotic stromal cells was examined in just one study (18). In that study, resveratrol treatment did not induce apoptosis in endometriotic stromal cells.

These findings encouraged us to investigate the gene expression of Bcl-2, Bax and Bcl-2/Bax ratio in eutopic (EuESCs) and ectopic (EESCs) endometrial stromal cells from patients with peritoneal endometriosis and endometrial stromal cells from non-endometriotic controls (CESCs).

## Materials and Methods

### Patient recruitment and sample collection

Forty women with peritoneal endometriosis and 15 women without endometriosis who underwent laparoscopic surgery or hysterectomy in the gynecology ward of Rassoul Akram Hospital, Tehran, Iran (January 2015 to April 2017) were enrolled in this study. All eligible women were of reproductive age (19–45 yr old), at the proliferative phase of the menstrual cycle, had normal menstrual cycles and had no history of hormonal treatment, pregnancy, breastfeeding, or using antioxidant supplements within the last three months before sampling. Patients with pelvic inflammatory disease, adenomyosis and suspected or ascertained diagnosis of malignancy were excluded. All patients with endometriosis were classified into stage III–IV according to the revised American Fertility Society system (19).

All experimental procedures were approved by Human Ethics Committees of the Iran University of Medical Sciences (No: IR.IUMS.REC.1395.28108) and signed informed consent was obtained from each participant before enrolling in the study.

Ectopic and eutopic endometrial samples were obtained through laparoscopic sampling and biopsy curette, respectively. In parallel, small fragment of the excised tissues were examined histologically for confirmation of endometriosis.

### Isolation of endometrial stromal cells (ESCs) and cell culture

Fresh endometriotic tissues were collected in sterile Dulbecco’s modified Eagle’s medium (DMEM) –F12 (Gibco, Grand Island, NY, USA) containing antibiotics and immediately transferred in cold chain to the laboratory, where minced into small pieces and digested at 37 °C for 120 min in DMEM/F-12 containing 100 U/ml penicillin and 100 μg/ml streptomycin, 2 mg/ml of type I collagenase (Sigma-Aldrich, St Louis, MO, USA) and 300 μg/ml of deoxynuclease I (Takara, Tokyo, Japan) with intermittent vortexing every 15 min (20). The obtained ESCs were then cultured and allowed to propagate not beyond passage three. Some samples were lost due to contamination or absence of enough cell growth especially in case of EESCs. Finally, cells from 13 eutopic and 11 ectopic endometrial tissues of endometriotic patients and 11 eutopic endometrial tissue from non-endometriotic patients were used in this study. Purity of isolated ESCs was assessed by flowcytometric analysis using a panel of antibodies. The ESCs were characterized as CD10^+^, CD73^+^, CD105^+^, CD44^+^, CD34^−^ and CD45^−^ cells.

### MTT assay

To investigate the safety dose of resveratrol, methyl thiazolyl tetrazolium *(*MTT) (Danesh Azma, Iran) assay was performed (21). Resveratrol (Sigma-Aldrich, St Louis, MO, USA) was dissolved in ethanol. Approximately 2×10^4^/well EuESCs from 4 endometriotic patients were seeded in duplicate in 96-well plates (SPL, Korea) and cultured for 24 h. After this time, EuESCs were treated in the presence or absence of resveratrol (12.5, 25, 50, 100, 200 and 400 μM). One h later, cells were stimulated with lipopolysaccharide (LPS) (100 ng/ml) (22, 23) as an inducer of apoptosis (24) and incubated in an incubator at 37 °C in a humidified, 5% CO_2_. The cytotoxic activity was determined after 24 and 48 h. Following 24 h incubation period, the medium was removed and 100 μl of fresh medium containing 12 mM MTT stock solution was added to all wells and plates were incubated for 4 h at 37 °C. Thereafter, medium was removed from the wells and 50 μl of MTT solvent was added to each well. After ensuring that all formazan crystals were dissolved, the absorbance was measured at 570 nm using a microplate ELISA reader (Bio-Tek Instruments Inc., Vermont, USA). The viability of the cells calculated using the following equation:

$$\begin{array}{l} \%\text{Viability}=[(\text{OD}\;\text{of}\;\text{treated}\;\text{cells}-\text{OD}\;\text{of}\;\text{MTT}\\ \text{solvent})/(\text{OD}\;\text{of}\;\text{untreated}\;\text{cells}-\text{OD}\;\text{of}\;\text{MTT}\\ \text{solvent})]\times 100 \end{array}$$

### Treatment of ESCs with resveratrol

In a pilot study, different concentrations of resveratrol ranging from 25–100 μM and also the time interval for the addition of LPS to the resveratrol-treated cells were set up. A collectively concentration of 100 μM for resveratrol was selected because this concentration has been shown to suppress cell proliferation or modulate biochemical processes in cancer cell lines (25). Thus, 30×10^4^/well ESCs were seeded in 24-well plates and after 3 h, cells were treated with 100 μM resveratrol or ethanol as vehicle control. One h later, 100 ng/ml LPS (23) was added to each well and cells were incubated for 6, 24 and 48 h.

### RNA extraction and cDNA synthesis

Total RNA from treated and untreated ESCs were extracted using the TRIZOL reagent (QIAGEN, Germany) according to the supplier’s protocol. The quantity of the extracted RNA was determined at an absorbance wavelength of 260/280 using a NanoDrop 2000 spectrophotometer (Thermo Fisher Scientific, Waltham, MA, USA). Reverse transcription of RNA (1 μg) was performed using a Revert Aid First Strand cDNA Synthesis Kit (Thermo Fisher Scientific, Waltham, MA, USA) according to the manufacturer’s instructions. The expressions of the genes of interest were normalized using glyceraldehyde 3-phosphate dehydrogenase (GAPDH) as a housekeeping gene. The following primers and annealing temperatures were used for amplification; Bcl-2: forward primer (F) 5′- ATCGCCCTGTG-GATGACTGAGT-3′ and reverse primer (R) 5′-GCCAGGAGAAATCAAACAGAGGC-3′ (62°C) (26); Bax: (F) 5′-CCCTTTT-GCTTCAGGGTTTCATCCA-3′ and (R) 5′-CTTGAGACACTCGCTCAGCTTCTTG-3′ (60°C) (27); GAPDH: (F) 5′-GCA CCG TCA AGG CTG AGA AC-3′ and (R) 5′-TGG TGA AGA CGC CAG TGG A-3′ (58°C) (28). The template and primer pairs were mixed with SYBER Green PCR Master Mix (BioFACT, South Korea) and real-time PCR was performed using a Rotor-Gene 3000 (Corbett Research, Sydney, Australia) with preliminary hold at 95 °C for 15 min, followed by 40 cycles of 95 °C for 20 sec, annealing and elongation for 40 sec and finally melting step was from 60 to 99 °C. Melting curve analysis was carried out to ensure the specificity of amplification.

### Statistical analysis

The statistical analysis of data was performed using GraphPad Prism software (ver. 6). The Kolmogorov–Smirnov distribution test was used in order to assess data normality. Based on the non-parametric distribution of data, Wilcoxon and Mann-Whitney U tests were used for comparison of variables within and between groups, respectively. For the comparison of more than two independent variables, Kruskal–Wallis test was used. In the case of significant differences between the study groups, differential effect was applied. *P*-value of <0.05 was considered as statistically significant.

## Results

### The effect of resveratrol on EuESCs viability

**Fig. 1:**
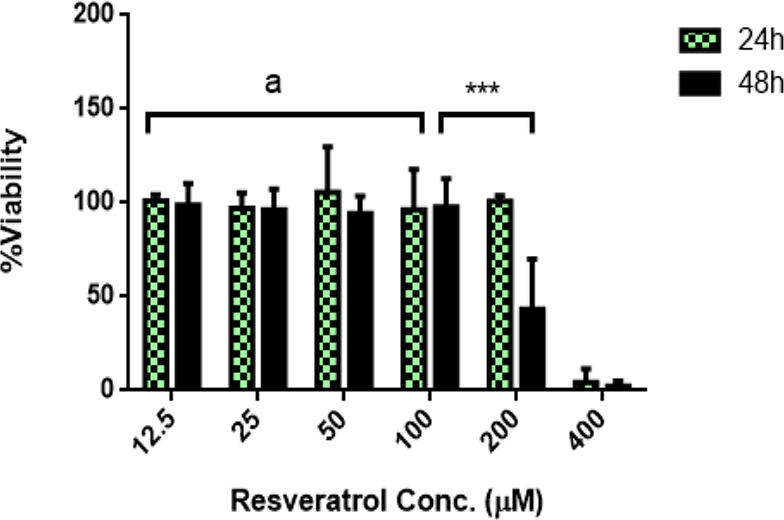
The effect of resveratrol on EuESCs viability. EuESCs were treated with and without resveratrol (12.5–400 μM) for 24 and 48 h in the presence of LPS (100 ng/ml). Cell viability was examined by MTT assays. Data were represented as medians (interquartile range) of two separate experiments. Statistical comparisons were made by an analysis of variance (two-way ANOVA), with a 95% confidence interval followed by Tukey post hoc test to identify significant differences between mean values of the target cells and the controls. In the figure, “a” indicates “no significant differences”; while “***” indicates “*P* < 0.001”. EuESCs, eutopic endometrial stromal cells; LPS, lipopolysaccharide; MTT, methylthiazolyl tetrazolium

The viability of EuESCs was determined by MTT assay. Cells were treated with resveratrol (12.5–400 μM) for 24 and 48 h. Resveratrol treatment with concentrations lower than 100 μM for up to 48 h had no effect on EuESCs viability while treatment of these cells with 200 μM of resveratrol for 48 h, reduced EuESCs viability to 50% (*P*> 0.001) (Fig. 1).

### The effect of resveratrol on Bcl-2 gene expression

**Fig. 2:**
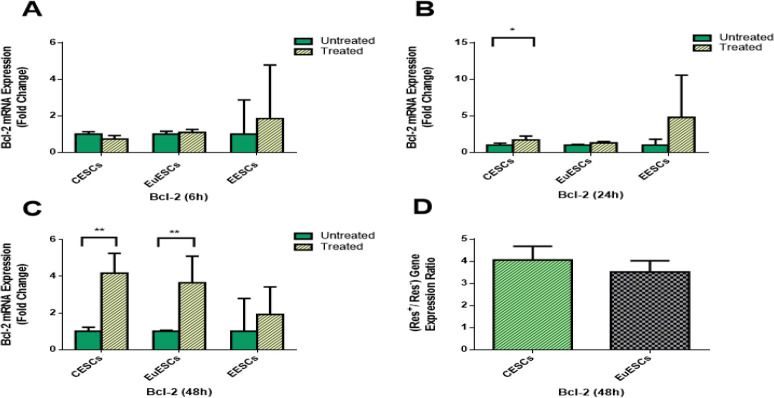
Quantitative RT-PCR gene expression of Bcl-2 in CESCs, EuESCs and EESCs treated with or without resveratrol for 6, 24 and 48 h. CESCs from non-endometriotic controls (n = 11), EuESCs (n = 13) and EESCs (n = 11) from endometriotic patients were cultured in the presence or absence of resveratrol for 6 (A), 24 (B) and 48 h (C). The bar graph indicates the fold change of Bcl-2 mRNA expression. (D) Differential effect of resveratrol on EuESCs and CESCs at 48 h were calculated as the ratio of Bcl-2 gene expression in the presence and absence of resveratrol. Data are expressed as means ± SEM. *P<0.05, **P<0.01. CESCs, control endometrial stromal cells; EuESCs, eutopic endometrial stromal cells; EESCs, ectopic endometrial stromal cells

As Fig 2 shows, resveratrol treatment increased Bcl-2 gene expression in CESCs at 24 (*P*<0.05; Fig. 2B) and 48 h (*P*<0.01; Fig. 2C) and in EuESCs at 48 h (*P*<0.01; Fig. 2C), but had no significant effects on the expression of this gene in EESCs. Furthermore, resveratrol treatment had no significant differential effect on increased Bcl-2 gene expression between EuESCs and CESCs at 48 h (Fig. 2D).

### The effect of resveratrol on Bax gene expression

**Fig. 3:**
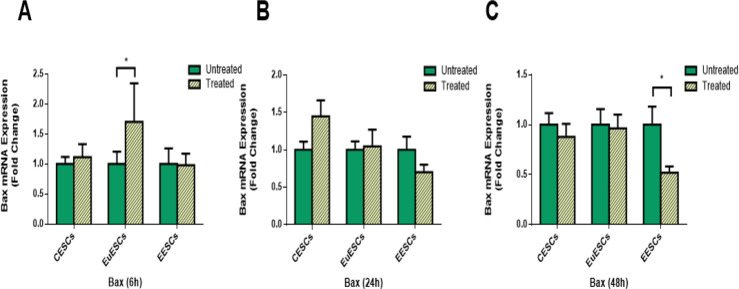
Quantitative RT-PCR gene expression of Bax in CESCs, EuESCs and EESCs treated with or without resveratrol for 6, 24 and 48 h. CESCs from non-endometriotic controls (n = 11), EuESCs (n = 13) and EESCs (n = 11) from endometriotic patients were cultured in the presence or absence of resveratrol for 6 (A), 24 (B) and 48 h (C). The bar graph indicates the fold change of Bax mRNA expression. Data are expressed as means ± SEM. Statistical comparisons were made by Wilcoxon matched-pairs signed rank test. *P < 0.05. CESCs, control endometrial stromal cells; EuESCs, eutopic endometrial stromal cells; EESCs, ectopic endometrial stromal cells

Resveratrol treatment increased Bax gene expression in EuESCs at 6 h (*P*<0.05; Fig. 3A) and decreased its expression in EESCs at 48 h (*P*<0.05; Fig. 3C).

### The effect of resveratrol on Bcl-2/Bax gene expression ratio

**Fig. 4:**
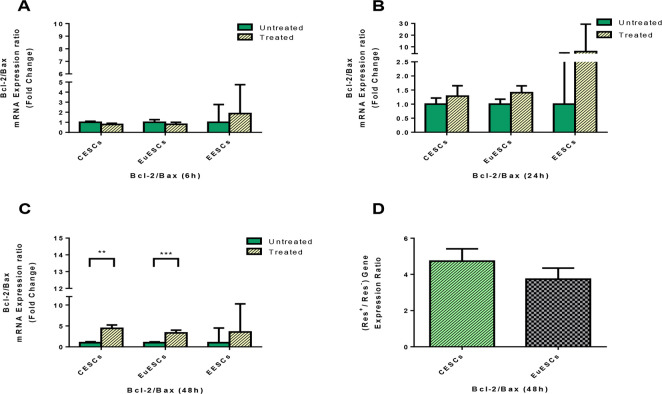
Quantitative RT-PCR gene expression of Bcl-2/Bax ratio in CESCs, EuESCs and EESCs treated with or without resveratrol for 6, 24 and 48 h. CESCs from non-endometriotic controls (n = 11), EuESCs (n = 13) and EESCs (n = 11) from endometriotic patients were cultured in the presence or absence of resveratrol for 6 (A), 24 (B) and 48 h (C). The bar graph indicates the fold change of Bcl-2/Bax mRNA expression ratio. (D) Differential effect of resveratrol on EuESCs and CESCs at 48 h were calculated as the ratio of Bcl-2/Bax gene expression in the presence and absence of resveratrol. Data were represented as mean ± SEM. **P < 0.01 and ***P ≤ 0.001. CESCs, control endometrial stromal cells; EuESCs, eutopic endometrial stromal cells; EESCs, ectopic endometrial stromal cells

Resveratrol treatment increased Bcl-2/Bax gene expression ratio in CESCs and EuESCs at 48 h (*P*<0.01 and *P*≤0.001 respectively; Fig. 4C) but had no significant effect on Bcl-2/Bax gene expression ratio in EESCs (Fig. 4). Besides, resveratrol treatment had no significant differential effect on increased Bcl-2/Bax gene expression ratio between EuESCs and CESCs at 48 h (Fig. 4D).

## Discussion

In this study, resveratrol treatment increased Bcl-2/Bax gene expression ratio in EuESCs and CESCs. To our knowledge, this is the first report evaluating the effects of resveratrol on apoptosis by investigating Bcl-2, Bax and Bcl-2/Bax gene expression ratio in ESCs. Resveratrol has been reported to exert a variety of biological effects, but the underlying mechanisms regarding apoptosis are not well understood. Recently, conflicting results were reported regarding induction of apoptosis by resveratrol in endometriosis (10, 11, 17, 18). Bruner-Tran et al. have recently demonstrated that resveratrol increased TUNEL staining of endometrial implants (10). Besides, BALB/c mice underwent surgical induction of endometriosis. Resveratrol treatment in this study, significantly increased apoptosis in the lesions (11). On the other hand, in a randomized study in a mouse model of endometriosis, resveratrol treatment had no effect on caspase-3-positive apoptotic cells in endometriotic lesions (17). Besides, resveratrol treatment did not induce apoptosis measured by annexin V-FITC in endometriotic stromal cells (18). On the other hand, in our study resveratrol treatment significantly increased Bcl-2/Bax gene expression ratio in EuESCs and CESCs.

One explanation for such differing results could be explained by possible differences in the methodology. In animal models of endometriosis, endometriotic lesions were induced differently. Some induced endometriotic lesions surgically using endometrial tissues from women without endometriosis (10) or used uterine tissue from donor mice into the peritoneal cavity of recipient mice without using endometriotic tissue of human origin (17). Endometrium from women with endometriosis and endometriotic implants have some major differences compared with the normal endometrium of non-endometriotic women (29). Furthermore, induction of apoptosis observed in mouse model of endometriosis, was not the direct result of resveratrol, but was secondary to the effect of resveratrol on suppression of vascularization (11,17) and invasiveness (10).

There is a plethora of evidence suggesting an upregulation of anti-apoptotic genes and reciprocal down-regulation of the genes regulating the apoptosis pathway in endometriotic stromal cells and resistance of these cells to apoptosis (30). Besides apoptosis, oxidative stress plays an important role in endometriosis (31). Presence of macrophages, iron or environmental contaminants in the peritoneal fluid of some women leading to oxidative stress, tissue growth and endometriosis (32). Furthermore, positive association between oxidative stress and endometriosis have been found in some studies (31). Increase in reactive oxygen species (ROS) production is associated with apoptosis (33). However, the magnitude of this effect will depend upon the cellular genetic background, the types and levels of the specific ROS involved and duration of the oxidative stress (34). On the other hand, many inhibitors of apoptosis have antioxidant activities or enhance cellular antioxidant defenses (35). Diets depleted of the antioxidants selenium and α-tocopherol were enhanced apoptosis and inhibited brain tumor growth in transgenic mice model (36). In our study resveratrol as a potent inhibitor of ROS production (37), increased Bcl-2/Bax gene expression ratio in EuESCs and CESCs. Similarly, in other studies resveratrol treatment attenuated oxidative stress-induced cell death in cultured rat pheochromocytoma (PC12) cells (38) and human neuroblastoma cell line SH-SY5Y (39).

Another explanation for our findings could be explained by the role played by growth factors as an epidermal growth factor (EGF) and other growth factors stimulate endometriotic stromal cell’s growth (40) and inhibit resveratrol’s action on both ERK1/2 activation and the induction of apoptosis via the p53 pathway in cancer cells (41). On the other hand, EESCs and EuESCs altered responses to apoptosis in our study could be explained by differences in the microenvironment and inflammatory condition of peritoneal cavity of endometriotic patients that affecting endometriotic cell’s responses to nutritional interventions (20, 26).

A key strength of this study is that we used a large sample of the study subjects, and all our patients were examined in the same phase of the menstrual cycle. However, there are some limitations in our study. First, it may be essential to evaluate resveratrol effect in combination with other antioxidants in a long period of time. The second limitation was that we did not assess protein expression of apoptotic molecules and apoptosis.

## Conclusion

Resveratrol treatment increased Bcl-2/Bax gene expression ratio in EuESCs and CESCs but had no effect on EESCs. Although apoptosis has an important role in pathophysiology of endometriosis, but further in vitro and in vivo studies are warranted to fully investigate resveratrol effects on cell proliferation, invasion, adhesion and other factors related to the pathogenesis of endometriosis.

## Ethical considerations

Ethical issues (Including plagiarism, informed consent, misconduct, data fabrication and/or falsification, double publication and/or submission, redundancy, etc.) have been completely observed by the authors.
